# Increased Oxidative Stress Response in Granulocytes from Older Patients with a Hip Fracture May Account for Slow Regeneration

**DOI:** 10.1155/2014/819847

**Published:** 2014-03-02

**Authors:** Zhiyong Wang, Sabrina Ehnert, Christoph Ihle, Lilianna Schyschka, Stefan Pscherer, Natascha C. Nussler, Karl F. Braun, Martijn Van Griensven, Guobin Wang, Rainer Burgkart, Ulrich Stöckle, Florian Gebhard, Helen Vester, Andreas K. Nussler

**Affiliations:** ^1^Traumatology, Klinikum rechts der Isar, Technische Universität München, Ismaninger Straße 22, 81675 München, Germany; ^2^Department of Surgery, Union Hospital, Tongji Medical College, Huazhong University of Science and Technology, Wuhan 430030, China; ^3^Siegfried Weller Institute for Trauma Research, BG Trauma Center Tübingen, Eberhard Karls University Tübingen, Schnarrenbergstraße 95, 72076 Tübingen, Germany; ^4^Department of Diabetology, Klinikum Traunstein, Cuno-Niggl-Straße 3, 83278 Traunstein, Germany; ^5^Department of Surgery, Neuperlach Hospital, Städtisches Klinikum München GmbH, Oskar-Maria-Graf-Ring 51, 81737 München, Germany; ^6^Clinic for Orthopedy, Klinikum rechts der Isar, Technische Universität München, Ismaninger Straße 22, 81675 München, Germany; ^7^Department of Trauma, Hand, Plastic and Reconstructive Surgery, Albert-Einstein University Ulm, Albert-Einstein-Allee 39, 89081 Ulm, Germany

## Abstract

Proximal femur fracture, a typical fracture of the elderly, is often associated with morbidity, reduced quality of life, impaired physical function and increased mortality. There exists evidence that responses of the hematopoietic microenvironment to fractures change with age. Therefore, we investigated oxidative stress markers and oxidative stress-related MAPK activation in granulocytes from the young and the elderly with and without fractured long bones. Lipid peroxidation levels were increased in the elderly controls and patients. Aged granulocytes were more sensitive towards oxidative stress induced damage than young granulocytes. This might be due to the basally increased expression of SOD-1 in the elderly, which was not further induced by fractures, as observed in young patients. This might be caused by an altered MAPK activation. In aged granulocytes basal p38 and JNK activities were increased and basal ERK1/2 activity was decreased. Following fracture, JNK activity decreased, while ERK1/2 and p38 activities increased in both age groups. Control experiments with HL60 cells revealed that the observed p38 activation depends strongly on age. Summarizing, we observed age-dependent changes in the oxidative stress response system of granulocytes after fractures, for example, altered MAPK activation and SOD-1 expression. This makes aged granulocytes vulnerable to the stress stimuli of the fracture and following surgery.

## 1. Introduction

Proximal femur fracture is a typical fracture of the elderly who frequently exhibit a lower bone mineral density, like, for example, osteopenia and osteoporosis, as well as muscle atrophy [1]. All over the world, approximately 2.6 million patients suffer from a hip fracture each year. Due to the anticipated demographic changes, this number is expected to be doubled in 40 years [2, 3]. Despite the major progresses regarding surgical procedures, hip fractures are still a major public health concern. High postoperative complication rates negatively affect the general outcome as well as the quality of life and cause high costs for the health care system [2, 4]. However, little is known about the mechanisms influencing the recovery of the patients.

Changes in the microarchitecture and loss of bone substance may render the surgical treatment more complicated and delay fracture healing due to a disbalance of the function of osteoblasts and osteoclasts. However, these negative effects of bone remodeling do not provide sufficient explanation for the high rate of postoperative complications after hip fracture particularly in older patients [5]. The increased morbidity and mortality, for example, due to infections, in this subgroup of patients is more likely due to systemic changes which have negative effects on recovery [6]. Circulating cells of the immune system are directly exposed to the stress stimuli caused by the fracture and the following surgery. Thus, normally they should be able to respond to the external stress signal directly. In elderly patients, these responses are possibly impaired due to alterations of the immune system.

Granulocytes and monocyte derived macrophages constitute the first lines of defense against bacterial infections. After a trauma, neutrophils migrate to the site of injury within minutes, where mitogen-activated protein kinase (MAPK) signaling occurs in granulocytes as a concomitant circumstance to extracellular stimuli that affect the functions of the cell, like, for example, proliferation, differentiation, or apoptosis [7, 8].

MAPKs that have been reported to be involved in these processes are JNK, ERK1/2, and p38 [9–16]. These MAPKs are all directly affected by oxidative stress stimuli [7, 9]. Their activation may induce cellular defense mechanisms, for example, the expression of the antioxidative enzyme superoxide dismutase (SOD-1) which directly eliminates reactive oxygen species (ROS) by catalyzing the dismutation of superoxide to hydrogen peroxide and molecular oxygen [17].

Thus, the aim of the present study consisted in investigating oxidative stress levels, oxidative stress induced toxicity, and the related MAPK activation in granulocytes from young (<50 years of age) and elderly (>65 years of age) patients with fractures of the long bones and from healthy controls. This comparison should enable us to draw conclusions about alterations of the response of granulocytes to the stress stimuli of a fracture and following surgery in young and old patients.

## 2. Materials and Methods

Phosphate buffered saline, fetal calf serum, and RPMI1640 were purchased from PAA Laboratories GmbH (Pasching, Austria). Complete protease inhibitor was obtained from Roche (Mannheim, Germany). All other chemicals were purchased from Sigma (Munich, Germany).

### 2.1. Ethics Statement

Blood sampling was conducted in accordance with the Declaration of Helsinki (1964) and its amendments. The study protocol was approved by the hospital's Ethics Committee and informed consent was obtained from all subjects. Patients with chronic diseases were not included in the study.

### 2.2. Sample Population

45 mL venous blood (36 mL EDTA (ethylenediaminetetraacetic acid) blood and 9 mL serum blood) was collected from each patient. Two different age groups were studied: young patients aged less than 50 years and elderly patients aged over 65 years. In both age groups, we distinguished between healthy controls and patients with a fracture of the long bones. The study population consisted of 22 young healthy controls (YH), 23 old healthy controls (OH), 12 young patients (YF), and 19 old patients (OF). In both groups of patients, the blood sampling was performed within 6 h after the operative reposition of the fracture of one of the long bones of the lower extremity. Demographic data is summarized in Table 1.

### 2.3. Granulocyte Isolation

The granulocytes were isolated by MACS (magnetic cell separation) using CD15 magnetic microbeads according to the manufacturer's instructions (Miltenyi Biotec, Cologne, Germany) or by density gradient centrifugation as described in [18]. Immediately after their isolation, the granulocytes were lysed for Western blot analysis. Cell purity was over 95% as assessed by flow cytometry.

### 2.4. HL60 Cell Culture

The HL60 granulocyte cells were expanded in RMPI 1640 medium containing 10% fetal calf serum, 2 mM L-glutamine, 100 U/mL penicillin, and 100 *μ*g/mL streptomycin at 37°C and 5% CO_2_. For maturation culture medium was supplemented with 1% dimethylsulfoxide. Prior to the experiment, the cells were serum-starved overnight. After 1 h stimulation with culture medium containing 10% sera from young and old patients and controls, cells were lysed for Western blot analysis.

### 2.5. Quantification of Lipid Peroxidation to Assess Oxidative Stress Levels

In order to measure the oxidative stress, lipid peroxidation was determined through the malondialdehyde assay. Briefly, 15 *μ*L serum was incubated with 45 *μ*L thiobarbituric acid solution (0.33% thiobarbituric acid in 11.1% acetic acid) for 1 h at 100°C. The samples were diluted with distilled water and their fluorescence was measured immediately at an excitation/emission wavelength of 544/590 nm. Known concentrations of 1,1,3,3-tetraethoxypropane were used as a standard control.

### 2.6. Viability Measurement

The cells' viability was determined by resazurin conversion. Briefly, cells (~7∗10^5^ cells/well) were stimulated with various concentrations of H_2_O_2_ (0, 0.002, 0.004, 0.008, 0.016, 0.03, 0.06, 0.125, 0.25, 0.5, and 1%). 1/10 volume of a 0.025% (w/v) resazurin solution (in phosphate buffered saline) was added to the cells. After 1 h incubation at 37°C fluorescence was measured (ex/em = 544/590 nm) and corrected to background control (no cells). Viability is given as % of untreated control.

### 2.7. Western Blot Analysis

Cells were lysed in freshly prepared ice-cold RIPA buffer (50 mM tris(hydroxymethyl)-aminomethan, 250 mM NaCl, 2% Nonidet-P40, 2.5 mM EDTA, 0.1% sodium dodecyl sulfate (SDS), 0.5% deoxycholic acid, protease and phosphatase inhibitors, pH = 7.2). Protein concentration was determined by micro-Lowry [19]. 30 to 50 *μ*g total protein was separated by SDS-PAGE and transferred to nitrocellulose membranes (Roth, Karlsruhe, Germany). Unspecific binding sites were blocked with 5% bovine serum albumin in TBST solution (25 mM tris(hydroxymethyl)-aminomethan, 137 mM NaCl, 2.7 mM KCl, 0.05% Tween-20, pH = 7.4) for 1 h at RT. After overnight incubation with primary antibodies (Cell Signaling, Beverly, USA) diluted 1 : 1,000 in TBST at 4°C, membranes were incubated with the corresponding horseradish peroxidase-labeled secondary antibodies (1 : 10,000 in TBST) for 2 h at RT. Chemiluminescent signals were detected with X-ray films or a charge-coupled device camera (CCD camera). In order to compare specific signals on different blots, a uniform positive control (HEK293T cells) was included for each membrane. Signal intensities were determined by densitometry using the ImageJ software (NIH, Bethesda, USA) and normalized to the corresponding GAPDH signals.

### 2.8. Statistics

**Figure 1:**
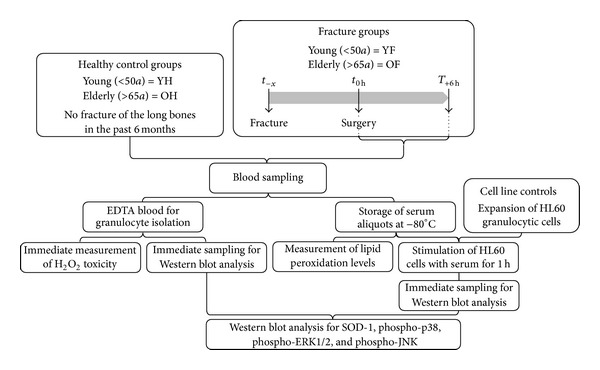
Overview on the experimental setup.

A flow diagram on the experimental setup is given in Figure 1. Results are expressed as mean ± SEM. The amount of donors (*N*) and replicates (*n*) is given separately for each experiment. Datasets were compared by one-way analysis of variance or by the Student's *t*-test (GraphPad Prism Software, LaJolla, CA, USA). *P* < 0.05 was taken as the minimum level of significance.

## 3. Results

### 3.1. The Elderly Show Increased Lipid Peroxidation Levels Representing Elevated Oxidative Stress Levels

**Figure 2:**
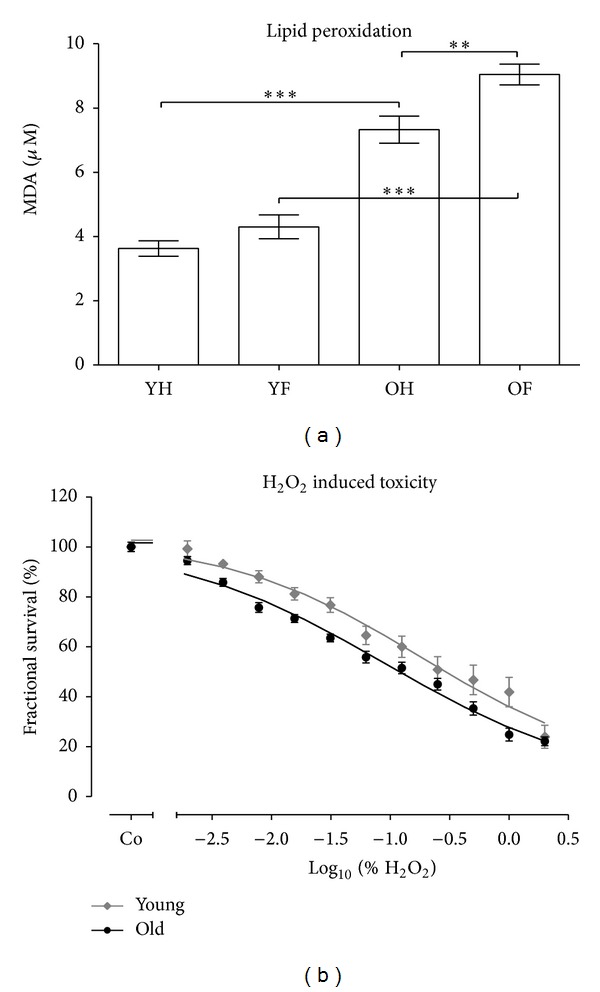
Aged granulocytes are more sensitive towards H_2_O_2_ induced toxicity as young granulocytes. (a) Levels of oxidative stress were determined by measuring lipid peroxidation in sera of young controls (YH), young patients with a fracture of the long bones (YF), elderly controls (OH), and elderly patients with a fracture of the long bones (OF). *N* = 10 and *n* = 2 per group. **P > 0.01 and ***P < 0.001 as determined by one-way ANOVA. (b) Granulocytes from young controls (*N* = 5, *n* = 4) and elderly controls (*N* = 5, *n* = 4) were stimulated with various concentrations of H_2_O_2_ (0, 0.002, 0.004, 0.008, 0.016, 0.03, 0.06, 0.125, 0.25, 0.5, and 1%). After 1 h viability was assessed by resazurin conversion. EC_50/young_ = 0.18% and EC_50/old_ = 0.09% (*P* < 0.05 as determined by Student's *t*-test).

In order to determine the levels of oxidative stress in the donors, the lipid peroxidation in the serum (*N* = 10/group, *n* = 2) was measured. The levels of basal lipid peroxidation in the older controls were approximately twice as high as those of young controls (*P* < 0.001). Interestingly, after the occurrence of a fracture a significant increase of about 25% of the lipid peroxidation was detected only in the older patients. Young patients with a fracture of the long bone showed no statistic increase in lipid peroxidation (Figure 2(a)).

### 3.2. Viability Measurement Indicates That Aged Granulocytes Are More Sensitive Towards H_2_O_2_ Induced Toxicity as Compared to Young Granulocytes

In order to investigate whether the increased oxidative stress affects the viability of granulocytes, we stimulated granulocytes from young and elderly controls with various concentrations of H_2_O_2_ (0, 0.002, 0.004, 0.008, 0.016, 0.03, 0.06, 0.125, 0.25, 0.5, and 1%). After 1 h viability was assessed by resazurin conversion. Granulocytes from elderly controls (*N* = 5, *n* = 4) were more sensitive towards H_2_O_2_ induced toxicity than granulocytes from young controls (*N* = 5, *n* = 4), represented by an EC_50_ of 0.09% and 0.18%, respectively (*P* < 0.05) (Figure 2(b)).

### 3.3. Altered SOD-1 Expression in Aged Granulocytes Is Detected by Western Blot Analysis

**Figure 3:**
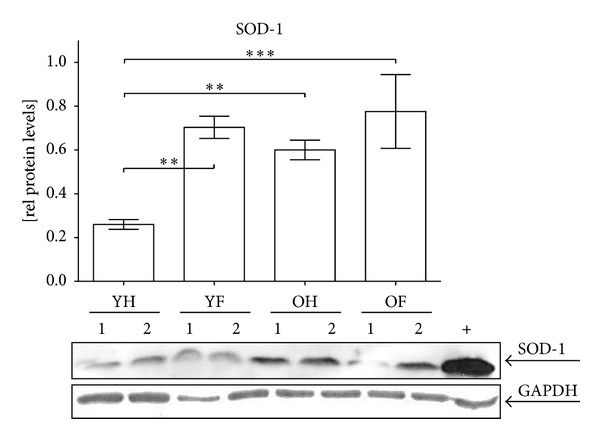
Altered SOD-1 expression in aged granulocytes. Representative Western blot for SOD-1. GAPDH was used as a loading control and for normalization. For intermembrane comparison HEK293T cells (+) were used. Densitometric analysis of all donors (YH: *N* = 22/YF: *N* = 12/OH: *N* = 23/OF: *N* = 19) was performed to determine the relative expression levels of SOD-1. **P > 0.01 and ***P < 0.001 as determined by one-way ANOVA.

In order to investigate why oxidative stress strongly affects aged granulocytes, we investigated the expression levels of SOD-1 by Western blot (Figure 3). Densitometric analysis revealed that the basal expression levels of SOD-1 were significantly elevated (2.2-fold) in the older controls in comparison to the young controls. After trauma and following surgery, the SOD-1 levels significantly increased in the young patients (2.7-fold). Interestingly, in older patients, the basally elevated expression levels of the SOD-1 did not further increase after trauma and following surgery.

### 3.4. Altered MAPKs Activation in Granulocytes of the Elderly Is Detected by Western Blot Analysis

**Figure 4:**
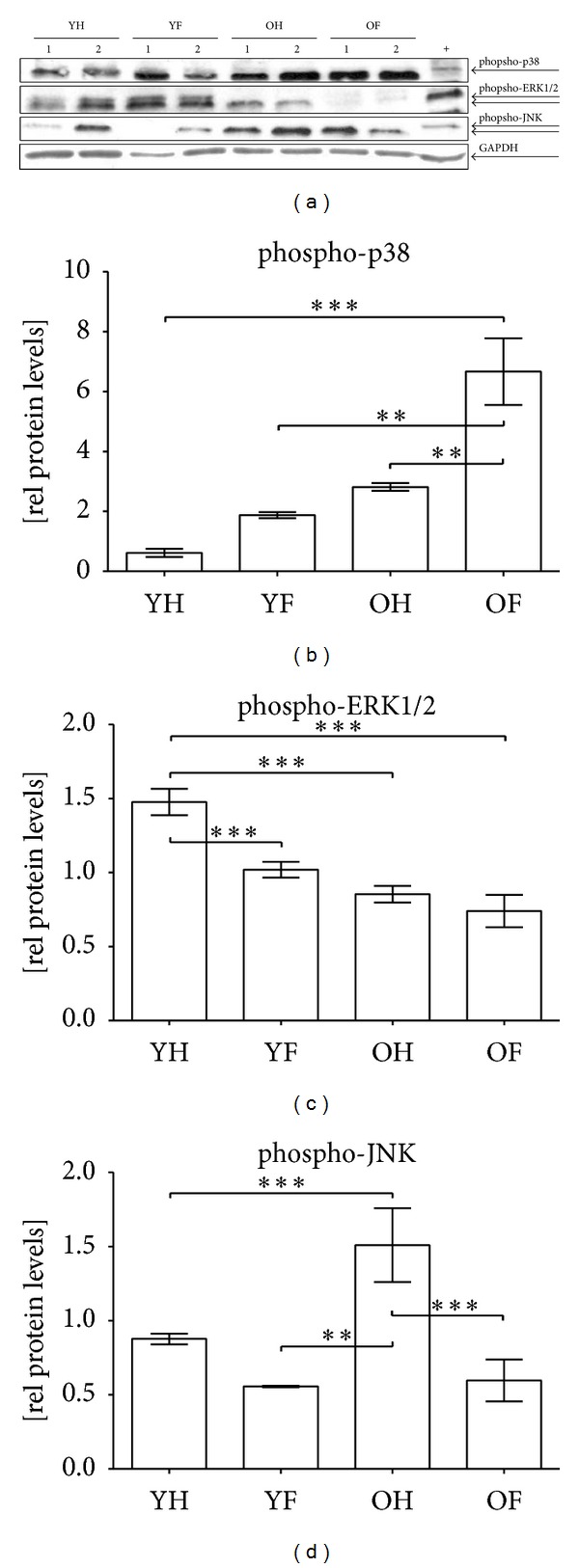
Altered activation of MAPKs in granulocytes of elderly patients and controls. (a) Representative Western blot for phospho-p38, phospho-ERK1/2, and phospho-JNK in granulocytes of young controls (YH), young patients with a fracture of the long bones (YF), elderly controls (OH), and elderly patients with a fracture of the long bones (OF). GAPDH was used as a loading control and for normalization. For intermembrane comparison HEK293T cells (+) were used. Densitometric analysis of all donors (YH: *N* = 22/YF: *N* = 12/OH: *N* = 23/OF: *N* = 19) was performed to determine the relative expression levels of (b) phospho-p38, (c) phospho-ERK1/2, and (d) phospho-JNK. **P > 0.01 and ***P < 0.001 as determined by one-way ANOVA.

In order to investigate whether the increased oxidative stress may affect the stress response of granulocytes, we investigated activation (phosphorylation) of the MAPKs p38, ERK1/2, and JNK by Western blot (Figure 4(a)). Basal activation of p38 was increased 4.5-fold in elderly controls as compared to young controls. After the occurrence of a fracture, a significant increase (2.5-fold) in the p38 activation was observed only in elderly patients. In young patients, however, the increase in p38 activity was not significant and did not reach the basal levels of older controls (Figure 4(b)). On the contrary, basal activation of ERK1/2 signaling was significantly decreased (about 40%) in elderly controls. After a fracture, ERK1/2 activation was further decreased only in young patients by about 30% (Figure 4(c)). Similar to p38 activation, basal JNK activation was significantly increased (1.9-fold) in elderly controls in comparison to young controls. After a fracture of the long bones, phospho-JNK levels decreased in both groups with the decrease being more pronounced in the group of the elderly (Figure 4(d)).

### 3.5. Western Blot Analysis Shows an Inverse p38 Activation in Serum Stimulated HL60 Cells in Comparison to Granulocytes

**Figure 5:**
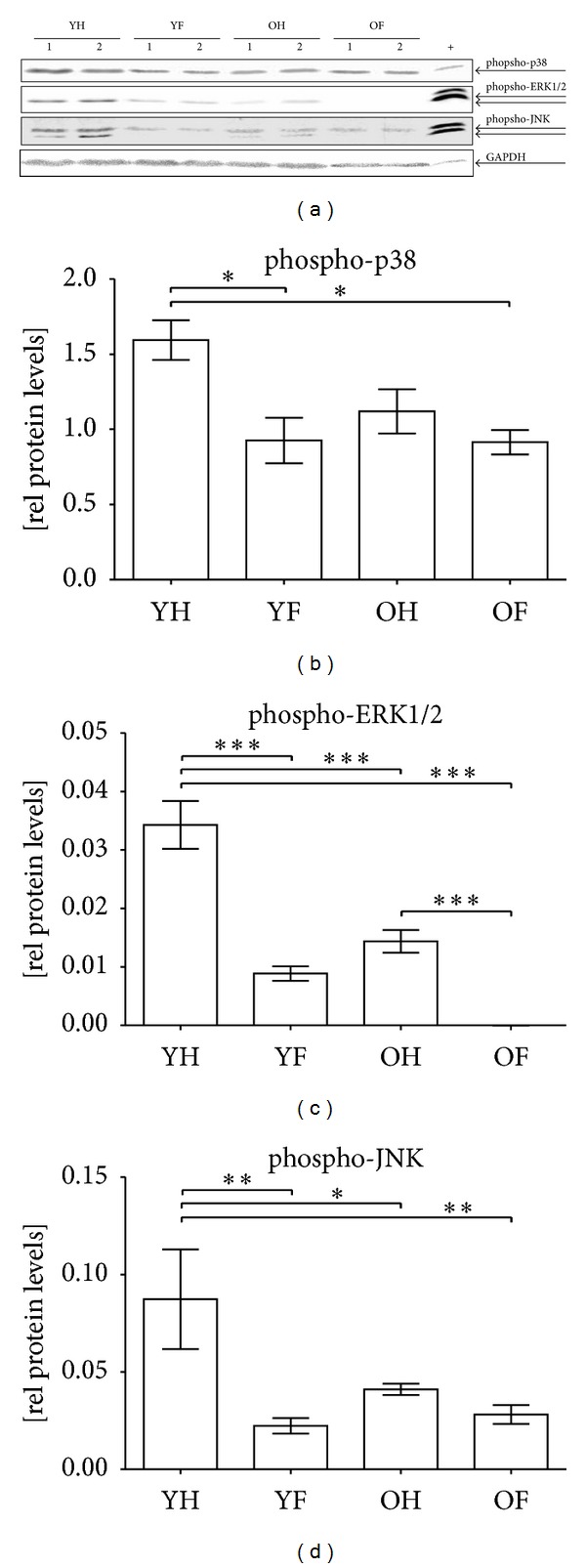
Inverted p38 activation in serum-conditioned HL60 cells in comparison to granulocytes. (a) Representative Western blot for phospho-p38, phospho-ERK1/2, and phospho-JNK in HL60 cells conditioned with serum of young controls (YH), young patients with a fracture of the long bones (YF), elderly controls (OH), and elderly patients with a fracture of the long bones (OF). GAPDH was used as a loading control and for normalization. For intermembrane comparison HEK293T cells (+) were used. Densitometric analysis of all samples (*N* = 10, *n* = 2/group) was performed to determine relative expression levels of (b) phospho-p38, (c) phospho-ERK1/2, and (d) phospho-JNK. *P > 0.05, **P > 0.01 and ***P < 0.001 as determined by one-way ANOVA.

In order to analyze whether the activation of MAPKs that has been observed in granulocytes is age-dependent or caused by the observed changes in the cellular microenvironment (serum), the activation of the three MAPKs in matured HL60 cells (considered to have the same age) cultured in the presence of the patients' sera was investigated (Figure 5(a)). Contrary to the granulocytes, basal activation of p38 was decreased (about 30%) in the HL60 cells stimulated with sera of the elderly controls in comparison to the HL60 cells stimulated with sera of the young controls. HL60 cells stimulated with patients' sera showed a significant decrease (2.5-fold) in p38 activation only in the young group (Figure 5(b)). Similar to the granulocytes, basal activation of ERK1/2 signaling was significantly decreased, by about 60%, in HL60 cells stimulated with sera of the elderly controls. In both age groups activation of ERK1/2 was reduced in HL60 cells stimulated with patients' sera as compared to stimulation with control sera (Figure 5(c)). On the contrary to granulocytes, basal JNK activation was significantly decreased, by 50%, in HL60 cells stimulated with sera of the elderly controls as compared to stimulation with control sera. The phospho-JNK levels further decreased in HL60 cells stimulated with patients' sera, with the decrease being more pronounced in the young group (Figure 5(d)).

## 4. Discussion

Hip fracture is a typical fracture of the elderly and its risk directly correlates with bone mineral density and bone strength [20, 21]. Thus, facing the current demographic changes with an increasing number of older people, the treatment of hip fractures represents an important problem for the public health systems [3, 4]. Despite all surgical advances over the past years, hip fractures are still accompanied by high complication rates, and therefore by an increased disability, morbidity, and mortality. While one-third of the patients require a higher level of long-term care, the in-hospital mortality accounts to almost 10% and the one-year mortality accounts for around 27% [22]. This high complication rate may be due to alterations in the immune system in the elderly, which render these patients prone to infections. Granulocytes are among the first lines of defense against bacterial infections. They are circulating immune cells and therefore they are directly exposed to the stress stimuli generated by the fracture and by the operative fracture fixation. Therefore, the aim of this project consisted in investigating the stress response of granulocytes obtained from young (<50 years of age) and elderly (>65 years of age) patients with a fracture of one of the long bones. Healthy individuals of both age groups served as control groups.

In the serum of older controls an increase in oxidative stress, represented by increased lipid peroxidation levels, was observed. ROS, particularly superoxide and its derivatives, induce an accumulation of oxidative damage to macromolecules in the cell, including proteins, lipids, and DNA, which in turn causes aging and eventually cell death [23, 24]. After the occurrence of a fracture and the following surgical reposition of the bone, the lipid peroxidation levels increased in the elderly group. Interestingly, in young patients, the levels of oxidative stress did not significantly increase after the occurrence of a fracture. This observation suggests that the young patients are able to actively reduce excessive amounts of ROS and reactive oxygen and nitrogen intermediated (ROI and RNI), for example, by expressing antioxidative enzymes. This is supported by our finding that aged granulocytes were more sensitive towards H_2_O_2_ induced cellular damage than granulocytes from young controls. SOD-1 is a free radical scavenger which is believed to act as the first line of defense against oxidative damage by catalyzing the conversion of superoxide radicals to hydrogen peroxide, which can then be reduced to water [25, 26]. In granulocytes of elderly controls, we observed increased basal SOD-1 levels. The increased basal SOD-1 levels are probably at maximum and cannot be further elevated after fractures. After the occurrence of a fracture, SOD-1 levels significantly increased only in the young patients. These findings confirm the oxidative stress theory of aging by Harman, in which he predicts that the imbalance between the formation of oxidative stress and antioxidant defense mechanisms results in a steady accumulation of oxidative damage in a variety of macromolecules [27].

The increase in oxidative stress in turn affects circulating immune cells that are directly exposed to the stimuli. Granulocytes migrate to the site of injury within minutes. In particular, the MAPKs JNK, p38, and ERK1/2 are directly affected by oxidative stress stimuli in these cells [7, 9].

Basal p38 signaling was increased in the granulocytes of the older controls. After the occurrence of a fracture, a considerable induction of p38 signaling was observed only in the older patients. After its activation, p38 initiates a signaling cascade that regulates the synthesis of a variety of proinflammatory mediators, like tumor necrosis factor *α* (TNF-*α*) [28], which was increased in the serum of older patients and controls. The basal JNK signaling was elevated in the elderly controls in a similar fashion to that of the p38 signaling. However, after the occurrence of a fracture, the activation of JNK dropped in young and older patients. Acute and transient activation of JNK is reported to induce cell proliferation and survival, while a prolonged and sustained activation of JNK may promote cell apoptosis [29] which is triggered by TNF-*α* and ROS [30] that are both elevated in the elderly patients and controls. Contrary to the p38 and JNK signaling, the basal ERK1/2 signaling was significantly lower in the elderly than in the young controls. After the occurrence of a fracture, ERK1/2 signaling was significantly lower in the young patients than in the young controls. However, in the elderly, this difference between the patients and the controls in the ERK1/2 signaling was much smaller. These findings are supported by the work of Schieven, in which it is shown that active p38 signaling may inhibit ERK1/2 signaling [28]. This is especially important as Larbi and coworkers were able to prove that the functional ERK1/2 signaling was able to inhibit granulocyte apoptosis which had been induced by Granulocyte-macrophage colony-stimulating factor (GM-CSF) [31]. Thus, granulocytes of older people have a higher susceptibility to apoptosis in the presence of elevated GM-CSF levels due to their impaired ERK1/2 signaling. Due to their increased rate of apoptosis in their granulocytes, elderly patients are more sensitive to infections following a trauma than young patients.

In order to investigate whether the observed alterations in the MAPK signaling are age-dependent or due to the observed changes in the microenvironment (cytokines, chemokines, and oxidative stress stimuli), we stimulated HL60 granulocytes (considered to have the same age) with patients' sera and investigated the activation of the MAPKs. ERK1/2 and JNK signaling were decreased in the elderly patients and controls and further decreased after a fracture analogously to the number of controls. However, contrary to the number of granulocytes, basal p38 signaling was decreased in the elderly controls and even further decreased after fracture. This observation suggests that p38 signaling is particularly prone to age-related changes. The finding of elevated p38 signaling in the granulocytes of the elderly patients is especially interesting as recent studies have demonstrated that it plays a key role in the persistent pain sensitization via neuronal and glial mechanisms [32–34]. Pain relief, before and after surgery, is one of the first priorities for patients with hip fractures, as adequate pain control may reduce complications, like, for example, cardiovascular events and restoring ambulation [6].

## 5. Summary

In summary, our data show an increase in oxidative stress levels in the elderly patients and controls. The strong upregulation of SOD-1 in granulocytes after fracture suggests that young patients are able to actively reduce ROS, ROI, and RNI, whereas in elderly patients, SOD-1 expression seems to have reached its limits already in a normal state. This might explain that aged granulocytes react more sensitively towards oxidative stress induced damage than the young granulocytes. The activation of MAPKs in the granulocytes indicates that young and old granulocytes react differently to the oxidative stress stimuli. p38 is especially age dependent. The shift in ERK1/2 and JNK signaling may sensitize the granulocytes of the elderly towards damage caused by oxidative stress stimuli.

## 6. Conclusion

Our data suggest that a balanced antioxidative response in granulocytes of elderly patients might protect them from additional stress stimuli, for example, fracture and following operation. A flavonoid rich diet is reported to induce the expression of antioxidative enzymes in various cell types [35–38]. However, this induction of the expression of antioxidative enzymes can only be reached, when flavonoids are consumed at high doses over a long period of time. This might even improve bone quality and thus reduce fracture risk, as the rat study with retinoic acid-induced bone loss from Orsolic and coworkers suggests [38]. However, in patients suffering from a fracture the first stress stimuli already occurred. Therefore, the clinic treatment can only be preventive against the second stress stimuli from the following operation, for example, by using drugs with strong antioxidative properties that can be applied in defined doses [35]. There have been first positive reports applying this strategy to prevent organ damage after abdominal surgery [39, 40]. However, if this treatment may also protect the immune cells has to be further evaluated in a controlled clinical trial.

## Figures and Tables

**Table 1:** Information about donors.

	Groups			
	YH	YF	OH	OF
Number of individuals	22	12	23	19
Age range (a)	24 to 38	22 to 47	71 to 90	69 to 99
Age median (a)	29.5	31	78	86
Number of male individuals	11	9	9	7
Number of female individuals	11	3	14	12

YH: young controls; YF: young patients with a fracture of the long bones; OH: elderly controls; OF: elderly patients with a fracture of the long bones.

## Abbreviations

ERK1/2: Extracellular signal-regulated kinases 1 and 2
GM-CSF: Granulocyte-macrophage colony-stimulating factor
JNK: c-Jun N-terminal kinase
MACS: Magnetic cell separation
MAPK: Mitogen-activated protein kinase
RNI: Reactive nitrogen intermediates
ROI: Reactive oxygen intermediates
ROS: Reactive oxygen species
SOD-1: Superoxide dismutase 1
TNF-*α*: Tumor necrosis factor *α*.
